# Knowledge, Utilization, and Associated Factors of Nonpneumatic Antishock Garments for Management of Postpartum Hemorrhage among Maternity Ward Health Care Professionals in South Wollo Zone Health Facilities, Ethiopia, 2021: A Cross-Sectional Study Design

**DOI:** 10.1155/2023/8247603

**Published:** 2023-01-14

**Authors:** Wondimnew Gashaw Kettema, Zenebe Tefera Ayele, Mandefro Assefaw Geremew, Kibir Temesgen Assefa, Sindu Ayalew Yimer, Atrsaw Dessie Liyew, Besfat Berihun Erega, Eyaya Habtie Dagnaw

**Affiliations:** ^1^Department of Midwifery, College of Medicine and Health Sciences, Wollo University, Dessie, Ethiopia; ^2^Department of Midwifery, Dessie Health Sciences College, Dessie, Ethiopia; ^3^Department of Midwifery, College of Medicine and Health Sciences, Debre Tabor University, Debre Tabor, Ethiopia

## Abstract

**Background:**

In 2017, approximately, 810 women died every day from preventable causes related to pregnancy and childbirth around the world. Obstetric hemorrhage, specifically postpartum hemorrhage, is the leading cause of preventable maternal mortality in the world. New strategies and technologies are needed to reduce the global public health epidemic of maternal mortality. However, nonpneumatic antishock garments were recently introduced and incorporated into teaching curriculums as a management modality for postpartum hemorrhage in Ethiopia. Therefore, this study assessed the knowledge, utilization and associated factors of nonpneumatic antishock garment among maternity ward healthcare professionals in the selected South Wollo zone health facilities, North West Ethiopia.

**Methods:**

An institutional-based cross-sectional study design was conducted from February 1 to April 30, 2021. A consecutive sampling technique was employed to collect the data. A self-administered semistructured English version questionnaire was used to collect the data. EPI-Info and SPSS were used for data entry and analysis, respectively. Bivariable and multivariable logistic regression analyses were used to analyze the association of nonpneumatic antishock garment utilization with independent variables.

**Results:**

A total of 244 maternity ward health care professionals participated. One hundred forty-six (59.8%) had a good knowledge of nonpneumatic antishock garments. About 110 (45.1%) of the participants have ever used it for the management of postpartum hemorrhage. Those having one nonpneumatic antishock garment (AOR = 2.7, 95% CI: 1.3, 5.5), two or more nonpneumatic antishock garments (AOR = 14.1, 5.7, 35.0), good knowledge (AOR = 5.2, 2.5, 10.7), and positive attitude (AOR = 2.5, 1.1, 5.7) and those who were receiving training (AOR = 2.2, 1.1, 4.4) at 95% CI were significantly associated with utilization of nonpneumatic antishock garments.

**Conclusion:**

The knowledge and utilization of nonpneumatic antishock garments for the management of postpartum hemorrhage were low. Those having more nonpneumatic antishock garments, good knowledge, and a positive attitude and those who received training were found to be significantly associated with nonpneumatic antishock garment utilization. The provision of training and availability of nonpneumatic antishock garments are the key actions to be taken to increase the utilization of nonpneumatic antishock garments.

## 1. Introduction

Postpartum hemorrhage (PPH) is defined as cumulative blood loss greater than 1000 ml accompanied by signs and symptoms of hypovolemia, following the birth of the baby up to the end of the puerperium [1]. Obstetric hemorrhage is the world's leading cause of maternal mortality, responsible for 27.1% of maternal mortality [2]. Postpartum hemorrhage is one of the preventable causes of maternal mortality with birth being attended by skilled health professionals [3]. A woman suffering from PPH can die within 2 hours unless she receives immediate and appropriate medical care [4].

For women suffering from uncontrollable PPH, a method to control the bleeding, reverse the shock, and stabilize the patient for safe transport to a comprehensive obstetric care facility could be lifesaving. One method to manage PPH is the use of a nonpneumatic antishock garment (NASG) [5].

Nonpneumatic antishock garment (NASG) is a first-aid device in the form of a lower body suit of articulated neoprene and Velcro segments that provides lower body circumferential counter pressure, which restores blood pressure to the core, thus treating hypovolemic shock. In 2006, the joint statement of the International Confederation of Midwives (ICM) and the International Federation of Gynecology and Obstetrics (FIGO) recommended research on antishock garments to reduce mortality among women suffering postpartum hemorrhage [6, 7].

This garment was used to maintain blood pressure during surgery. After undergoing numerous modifications, the suit was refined for use as an antigravity suit (G-suit) [8]. Further modification led to its use in the Vietnam War for resuscitating and stabilizing soldiers with traumatic injuries before and during transportation. The use of the garment for obstetrical hemorrhage in low-resource settings began in 2002 [6].

Currently, NASG is used to treat shock, resuscitate, stabilize, and prevent further bleeding in women with obstetric hemorrhage. The NASG reverses shock by returning blood from the legs and lower abdomen to the heart, lungs, and brain. This restores the woman's consciousness, pulse, and blood pressure [7, 9].

A nonpneumatic antishock garment was introduced in Ethiopia by the Clinton Health Access Initiative (CHAI) to make life-saving health interventions for women and newborns sustainable nationwide. However, it provides the equipment for only selected health facilities of four regions of Ethiopia that include Amhara, Oromo, Tigray, and Southern nation as well as the national people of Ethiopia[10].

About 295,000 women died during and following pregnancy and childbirth in 2017, and the estimated global maternal mortality ratio (MMR) in 2017 as 211 maternal deaths per 100,000 live births [3, 11]. The vast majority of these deaths (94%) occurred in low-resource settings and most could have been prevented [3]. Sub-Saharan Africa alone accounted for roughly two-thirds (196,000) of maternal deaths [3, 11]. According to the United Nations Maternal Mortality Estimation Inter-Agency Group (UN MMEIG) estimates of 2017, Ethiopia's MMR was 401 per 100,000 live births [11].

Delays in identifying hemorrhage, reaching tertiary care facilities, and receiving definitive care such as blood transfusions and surgeries are factors that led to maternal deaths in limited-resource settings [12]. During hemorrhage, applying NASG will decrease blood loss by half during an emergency hysterectomy for an intractable atonic uterus [4].

Therefore, postpartum hemorrhage management should be focused on the evaluation of the use of the NASG at the clinic level to determine if earlier application, before and during transport, will have a greater impact on decreasing adverse maternal outcomes [4].

Ethiopia targeted to achieve the maternal mortality ratio of sustainable development goal (SDG) by 2030 to less than 70 per 100,000 live births. Even though prevention of postpartum hemorrhage will reduce maternal mortality, interhealth facility distances in the South Wollo Zone from health center to hospital or hospital to hospital, ambulance travel may take more than two hours to reach the referred health institution. Hence, the result will motivate the government to supply the garments in healthcare settings and inform healthcare planners to develop policies that will ensure the availability of the nonpneumatic antishock garments in health centers and hospitals in Ethiopia. Therefore, this study was designed to assess knowledge, utilization and associated factors of NASG for the management of PPH among maternity ward health care professionals in selected South Wollo Zone health facilities.

## 2. Materials and Methods

### 2.1. Study Design, Area, and Period

An institutional-based cross-sectional study design was conducted on South Wollo Zone selected health facilities from February 1 to April 30, 2021. The administrative centre of the South Wollo Zone is Dessie town, which is located 401 kilometers north of Addis Ababa. Based on the 2007 Census conducted by the central statistical agency (CSA) of Ethiopia, South Wollo zone has a total population of 2,518,862, of whom 1,248,698 are men and 1,270,164 are women. South Wollo zone has 21 district woredas, 130 health centers, and 11 hospitals [13, 14]. According to the South Wollo zone reproductive health officer's report, training on NASG was given to selected hospital and woreda town health center labor and delivery health care professionals. NASG was only available in woreda town health centers and hospitals [15].

### 2.2. Source/Target Population

All maternity ward healthcare professionals who are working in South Wollo Zone health facilities were considered target population.

### 2.3. Study Population

All maternity ward healthcare professionals in the selected South Wollo Zone health facilities were available during the study period.

### 2.4. Inclusion Criteria

All maternity ward healthcare professionals who are working in the selected South Wollo Zone health facilities will be included.

### 2.5. Exclusion Criteria

Healthcare professionals who are on annual leave, sick leave, or maternity leave will be excluded.

### 2.6. Sample Size Determination

Sample size was taken from the highest sample by comparing the sample size of the three specific objectives. The first and second specific objectives were calculated by a single proportion formula. The proportion of healthcare professionals who have good knowledge and utilize on NASG study done in Jimma Zone hospitals were 59.5% and 36.2%, respectively [16], with 95% confidence level, 5% margin of error, and 10% nonresponse rate. We obtain 406 and 391 for the first and second objectives, respectively. Sample size for the third objective was calculated by Epi-info StatCalc, which gives 77. However, the total health care professionals in South woreda health centers and hospitals were estimated to be 267. Therefore, the final sample size is all health professionals in selected health facilities (census).

### 2.7. Sampling Procedure

NASG is only provided to those health facilities that have a higher case flow for maternal and child health care. Since the calculated sample size is higher than the target population, an entire population survey was employed to collect the data from 23 health centers and 11 hospitals.

### 2.8. Dependent Variables

Knowledge and utilization of NASG are dependent variables.

### 2.9. Independent Variables

Sociodemographic characteristics (age, sex, marital status, religion, ethnicity, professional qualification, and work experience) and attitude of the respondent on NASG are independent variables.

### 2.10. Operational Definition and Definition of Terms

Knowledge scale-respondent score of total knowledge questions of those below 50% were graded as having “poor knowledge,” while those who score 50.0% and above were graded as having good knowledge [16, 17].

Attitude scale-attitude of a respondent is considered “positive attitude” if the percentage score is 50% and above and is considered “negative attitude” if the respondent scores less than 50% [16].

Utilization of nonpneumatic antishock garment (NASG) is measured based on the response to the question of whether healthcare professionals used NASG for the management of postpartum hemorrhage at least one time [16].

### 2.11. Data Collection Tools and Procedures

Data were collected using a semistructured self-administered questionnaire which was adapted from similar studies [16–23]. The tool was prepared in English, which consisted of items like sociodemographic characteristics, knowledge about NASG, attitude toward NASG, and NASG utilization. One day of training was dedicated for learning the aim of the study, procedures, and data collection technique for four BSc midwifery professional data collectors and two MSc midwifery supervisors.

### 2.12. Data Quality Control

A pretest was conducted on 20 labor and delivery ward healthcare professionals in North Wollo health facilities prior to the actual study. Based on pretest, necessary adjustment to the data collection tools was made. Training was given to four data collectors and two supervisors. The supervisor supervised data collectors. At the end of each day, the questionnaires were reviewed and checked for completeness, accuracy, and consistency by the supervisor and investigator, and corrective discussion was conducted with all the research team members.

### 2.13. Data Processing and Analysis

The data were entered into a computer using Epidemiological Information (EPI-Info) version 7 software and exported to SPSS 23 (statistical packages for social science version 23) for analysis. The results were presented in percentages, means, tables, and charts. Data were analyzed using descriptive statistics and bivariable and multivariable logistic regression analyses.

A bivariable logistic regression model was used to identify factors associated with the outcome variable. A multivariable logistic regression model was fitted to control the possible effect of confounders, and finally, the independent variables which have an association with the outcome variable will be identified based on the odds ratio (OR), with a 95% confidence interval and *p* value less than 0.05. In the binary logistic regression, the variables associated with crude odds ratio (COR) with a *p* value <0.2 will be entered into the multivariable model. Model fitness was checked using Hosmer and Lemeshow's goodness of fit test, which gives 0.1.

### 2.14. Ethical Consideration

Ethical clearance was obtained from the Institutional Review Board (IRB) of Wollo University. Then, a formal cooperation letter was obtained from each health facility. Finally, written consent was obtained from the participants. Participants were informed that they could refuse or discontinue participation at any time they wanted. Confidentiality of the participant was assured by participants not including name or any identity.

## 3. Result

### 3.1. Sociodemographic Characteristics

A total of 244 maternity ward healthcare professionals participated, which gives a response rate of 94.9%. One hundred fifty-one (61.9%) of the participants were from hospitals. The mean age of participants was 27.7 years old with a standard deviation (SD) of ±4.1. More than half (61.1%) of the respondents were in the age group of 25–29 years old. One hundred thirty-five (55.3%) of the respondents were male. About one hundred eleven (45.5%) of the respondents were diploma midwife holders (Table 1).

### 3.2. Knowledge of Respondents on Nonpneumatic Antishock Garment (NASG)

One out of eight (13.5%) had never heard about a nonpneumatic antishock garment. The most common first source of information was health institutions as they used management modality (39.3%). Among those who have ever heard about NASG, 37% of the respondents correctly know what NASG looks like and more than half (57.3%) of the respondents mentioned that NASG has six segments. Regarding the function of nonpneumatic antishock garments, 140 (66.4%) mentioned that the nonpneumatic antishock garment prevents shock (Table 2).

**Figure 1:**
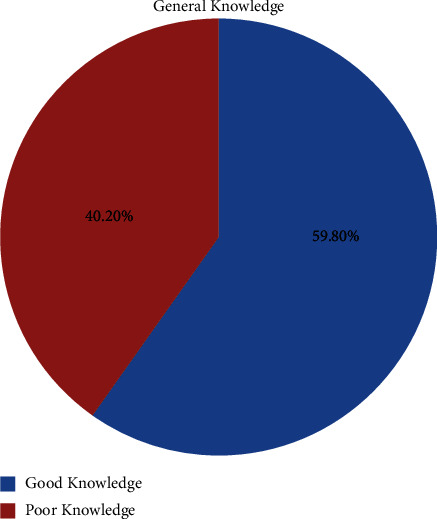
General knowledge of NASG for management of PPH among maternity ward health care professionals in selected South Wollo zone health facilities, North West Ethiopia, 2021.

About 59.8% of respondents had good knowledge of nonpneumatic antishock garments (Figure 1).

### 3.3. Attitude of Respondents toward Nonpneumatic Antishock Garment (NASG)

One hundred fifty-eight (64.8%) of the respondents agreed that NASG is necessary for the management of PPH in all settings. One hundred twenty-nine (52.9%) of the respondents agreed that an antishock garment is only beneficial to people in rural areas/primary care settings. One hundred fifty-six (64.0%) of the respondents agreed that the garment is only meant to be applied by healthcare professionals (Table 3). Regarding total attitude toward NASG, more than two-thirds (70.1%) had a positive attitude.

### 3.4. Utilization of Nonpneumatic Antishock Garment (NASG)

**Figure 2:**
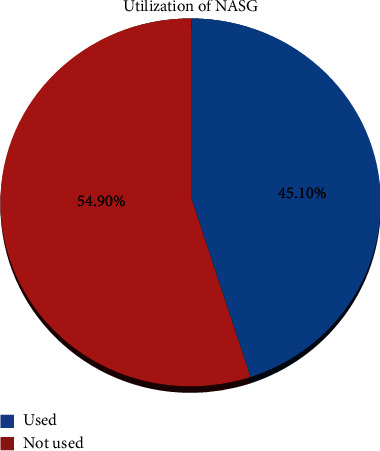
Utilization of NASG for management PPH among maternity ward health care professionals in South Wollo zone health facilities, North West Ethiopia, 2021.

One hundred ten (45.1%) of the participants have used NASG for the management of PPH (Figure 2).

Lack of experience 54 (40.3%) and not knowing about NASG 48(35.8%) are the two most common reasons for not utilizing NASG. Less than two-thirds (29.9%) of the participants have received training on NASG. Only near to half (50.4%) of the participants said NASG is available in their health facility (Table 4).

### 3.5. Factors Associated with Utilization of NASG for Management of PPH

On bivariable analysis, a hospital employee, a BSc midwife, those receiving training, those having a higher number of NASG, those having good knowledge, and those having a positive attitude were found significantly associated with increased NASG utilization for the management of PPH at a *p* value of 0.2. Then, a multivariable analysis was used to assess the net effect of those predictor variables.

Having training, good knowledge, a positive attitude, presence of one NASG, and presence of two and above NASG were found to be significantly associated with increased NASG utilization for the management of PPH multivariable logistic regression analysis (Table 5).

## 4. Discussion

According to this research finding, 59.8% of the participants have good knowledge regarding a NASG. This finding was in line with the finding of Jimma zone Southwest of Ethiopia which was 59.5% [16]. On the contrary, it is lower than the study findings in Specialist Hospital Sokoto, Sokoto State Nigeria, and the central hospital of Benin city which were 64% and 72.7%, respectively [17, 20]. The possible explanation for the discrepancy might be the number of health institutions included and the availability of NASG, where health professionals work. Furthermore, those comparison health facilities may have a higher level of academic qualification and sophisticated materials are more available that can lead to observing NASG. However, this finding is higher than the finding of midwives' knowledge of Ondo state selected hospitals, which was 45.8% [21]. This discrepancy might be due to the study participant's academic qualifications and time gap.

This study showed that 45.1% of the participants utilized NASG for the management of PPH. This finding is in line with the findings of the selected healthcare facilities in Bayelsa state, Nigeria, which was 46.4% [23]. This finding is higher than the studies conducted in Nigeria Ondo state selected hospitals, Nigeria, which was 14.1% [19], Ogun state hospitals, which was 22.7% [24], Ibadan University College Hospital, which was 35% [22], Benin City Central Hospital, which was 42% [17], and Ethiopia Jimma zone public hospital, which was 36.2% [16]. On contrary, this finding is lower than the study conducted in Bayelsa state, Nigeria, which was 52.5% [18]. This discrepancy might be due to the availability of NASG, the incorporation of healthcare professionals who are not the first point of contact for women during labor like nurses in medical ward, study participants' educational qualifications, and the non-inclusiveness of NASG as a management protocol of PPH. Furthermore, in some previous studies, NASG is only available in less than half of the study participants. Therefore, the nonavailability of NASG decreases the utilization of NASG.

Those professionals working in health facilities that have the garment have a better probability of utilizing NASG for the management of PPH. Healthcare professionals having one NASG in their health facility were 2.7 times more likely to utilize NASG for the management of PPH as compared to those healthcare professionals who have no NASG in their health facility (AOR = 2.7, 1.3–5.5). Healthcare professionals having two or more NASG in their health facility were 14.1 times more likely to utilize NASG for the management of PPH as compared to those healthcare professionals who have no NASG in their health facility (AOR = 14.1, 5.7–35.0). This finding is supported by the results of research studies conducted in Jimma zone public hospitals, Ethiopia, and selected hospitals of Ondo state, Nigeria [16, 19]. The possible reason might be due to the fact that the availability of the garment indicates NASG is one of the protocols for the management of PPH. In addition, increasing the NASG number allows utilizing the clean garment when the soaked garment is present.

Those having good knowledge were 5.2 times more likely to utilize NASG for the management of PPH as compared to their counterparts (AOR = 5.2, 2.5–10.7). This finding is in line with the results of research studies conducted in Nigerian government hospitals in Ogun State, Benin City, Edo State, and Jimma Zone public hospitals, Ethiopia [16, 17, 24]. The possible reason for having good knowledge associated with increased NASG utilization might be that knowledge is one of the prerequisites to applying NASG.

Those having a positive attitude toward NASG were 2.5 times more likely to utilize NASG for the management of PPH as compared to their counterparts (AOR = 2.5, 1.1–5.7). This is in line with the findings of Jimma Zone public hospitals, Ethiopia [16]. The possible reason might be that having a better attitude increases the intention to use NASG for the management of PPH.

Those who received training on NASG were 2.2 times more likely to utilize NASG for the management of PPH as compared to their counterparts (AOR = 2.2, 1.1–4.4). This is supported by the finding of Jimma Zone public hospitals in Ethiopia [16]. The possible explanation might be that NASG was not part of the obstetrics curriculum as a management protocol for PPH before four years in Ethiopian context, but it is only provided by training for health professionals.

### 4.1. Strength and Limitations of the Study

The study considers only healthcare professionals working in the labor and delivery ward, which shows a more precise result. Recall and social desirability bias on availability of NASG, utilization, and training on NASG are the limitations of the study.

## 5. Conclusion

The study showed that the knowledge of labor and delivery ward healthcare professionals on NASG for the management of PPH was low. The study revealed that utilization of NASG for the management of PPH was found to be low. Having good knowledge, more NASG, a positive attitude, and receiving training are found to be positive predictor variables for the utilization of NASG for the management of PPH. The researchers would like to recommend that the Amhara regional health bureau and the South Wollo zonal health office increase NASG availability at each public health facility by purchasing NASG like other medical equipment. It is better to provide NASG training for health professionals.

## Figures and Tables

**Table 1:** Socio-demographic characteristics and type of health facility of maternity ward healthcare professionals in South Wollo zone health facilities, North West Ethiopia, 2021.

Variables		Frequency	Percentage
Health facility category	Hospital	151	61.9
	Health center	93	38.1

Age	20–24	42	17.2
	25–29	149	61.1
	30–34	33	13.5
	35–39	12	4.9
	40–44	8	3.3

Sex	Male	135	55.3
	Female	109	44.7

Marital status	Single	125	51.2
	Married	113	46.3
	Widowed/divorced/separated	6	2.5

Ethnicity	Amhara	242	99.2
	Other^a^	2	0.8

Religion	Orthodox	160	65.6
	Muslim	80	32.8
	Protestant	4	1.6

Professional qualification	Diploma midwife	111	45.5
	BSc midwife	101	41.4
	Emergency surgeon	15	6.1
	Other profession^b^	17	7.0

Years of experience in a health profession (years)	1–5	184	75.4
	6–10	48	19.6
	11–15	6	2.5
	16–20	6	2.5

^a^ includes Oromia and South Nation and Nationalities' People (SNNP). ^b^ includes general practitioner (GP), BSc nurse, public health.

**Table 2:** Knowledge on NASG among maternity ward healthcare professionals in selected South Wollo Zone health facilities, North West Ethiopia, 2021.

Variables	Category	Frequency	Percentage
Have ever heard about NASG	Yes	211	86.5
	No	33	13.5

NASG looks like (*N* = 211)	Bottom half of suit	78	37.0
	Gown	28	13.2
	Trouser	105	49.8

The first source of information about NASG (*N* = 211)	Training	31	14.7
	Health institutions as they used management modality	83	39.3
	College/university education	63	29.9
	Internet	34	16.1

Number of segment NASG has (*N* = 211)	Four	54	25.6
	Six	121	57.3
	Eight	32	15.2
	Nine	4	1.9

The function of NASG (*N* = 211)^c^	Prevents shock	140	66.4
	Stabilizes the women in shock	101	47.9
	Reverses shock	75	35.5
	Decreases blood loss	126	59.7
	Compresses blood vessels	126	59.7
	Increases blood flow to vital organs	127	60.2
	I do not know	5	2.4

Indications for NASG application (*N* = 211)^c^	Postpartum hemorrhage	201	95.3
	Shock due to ectopic pregnancy	84	39.8
	Post-cesarean hemorrhage	104	49.3
	Shock due to trauma with injury/hemorrhage below the diaphragm	110	52.1
	I do not know	5	2.4

NASG applied for women with PPH (*N* = 211)^c^	Blood loss >750 ml	138	65.4
	Systolic blood pressure <90 mmHg	132	62.6
	Pulse >110 bpm	135	64.0
	I do not know	9	4.3

Contraindications for use of NASG (*N* = 211)^c^	Viable fetus in-utero	172	81.5
	Pulmonary edema	122	57.8
	Bleeding above the diaphragm	120	56.9
	Congestive heart failure due to mitral stenosis	128	60.7
	Dyspnea	83	39.3
	I do not know	15	7.1

How to apply NASG? (*N* = 211)	Start at the ankle and proceed up to the umbilicus	170	80.6
	Start at the umbilicus then proceed to the ankle	21	10.0
	Start at any segment	20	9.5

When to remove NASG? (*N* = 211)^c^	Estimated blood loss decreased to <50 ml/hr	93	44.1
	Hemoglobin level is >7 or hematocrit is >20%	106	50.2
	Pulse <100 bpm	130	61.6
	Systolic BP 90 mmHg or greater	114	54.0
	The woman is conscious and aware	107	50.7
	I do not know	16	7.6

“Rule of 20 cautions for NASG removal” means (*N* = 211)	The time interval between the removal of successive segments	92	43.6
	Blood pressure and pulse require reapply of segment/s when BP falls by 20 mmHg or pulse increases by 20 BPM respectively	119	56.4

^c-^ indicates multiple responses possible.

**Table 3:** Attitude toward NASG among maternity ward healthcare professionals in South Wollo Zone health facilities, Northwest Ethiopia, 2021.

Variables	Agree	Neutral	Disagree
	No (%)	No (%)	No (%)
NASG is necessary for the management of PPH in all settings	158 (64.8)	51 (20.9)	35 (14.3)
NASG can be used along with standard treatment protocols for PPH	185 (75.8)	42 (17.2)	17 (7.0)
NASG can be applied with minimum procedures in a short period	173 (70.9)	44 (18.0)	27 (11.1)
Removal of NASG requires a lot of procedures that take time	132 (54.1)	49 (20.1)	63 (25.8)
Antishock garment is only beneficial to people in rural areas/primary care settings	129 (52.9)	47 (19.3)	68 (27.8)
Manual removal of the placenta is possible with NASG in place	114 (46.7)	47 (19.3)	83 (34.0)
Antishock garment is effective in patients with cervical lacerations	89 (36.4)	48 (19.7)	107 (43.9)
The garment should be a must in every healthcare facility that has maternity services	179 (73.3)	39 (16.0)	26 (10.7)
The garment is only meant to be applied by healthcare professionals	156 (64.0)	47 (19.3)	41 (16.7)
The garment can transmit HIV to patients	38 (15.6)	47 (19.3)	159 (65.1)

Regarding total attitude toward NASG, more than two-thirds (70.1%) had a positive attitude.

**Table 4:** Utilization of NASG for management of PPH among maternity ward healthcare professionals in South Wollo zone health facilities, Northwest Ethiopia, 2021.

Variables		Frequency	Percentage
Received training on the use of NASG	Yes	73	29.9
	No	171	70.1

NASG is available at a health facility	Yes	123	50.4
	No	65	26.6
	I do not know	56	23.0

Number of NASG available (*N* = 123)	One	70	56.9
	Two and above	53	43.1

Ever used NASG in the management of PPH	Yes	110	45.1
	No	134	54.9

Reason/s for not utilizing NASG for PPH (*N* = 134)^d^	Availability of other methods	34	25.4
	Effective management of the third stage of labor	45	33.6
	Lack of experience	54	40.3
	Do not know about NASG	48	35.8
	The garment is not available	36	26.9

Will they use it if they know how to use it?	Yes	205	84.0
	No	39	16.0

Will they use it if a garment is available? (*N* = 121)	Yes	88	72.0
	No	33	28.0

^d-^ Multiple responses are possible.

**Table 5:** Bivariable and multivariate analyses of factors associated with utilization of NASG for management of PPH among maternity ward healthcare professionals in South Wollo Zone health facilities, Northwest Ethiopia, 2021.

Variables	*Ever used NASG*		COR (95%)	AOR (95%)
	Yes	No		
*Health facility category*				
Health center	33	60	1	1
Hospital	77	74	1.9(1.1–3.2)^*∗*^	1.1 (0.6–2.3)
*Professional qualification*				
Diploma midwife	41	70	1	1
BSc midwife	52	49	1.8(1.1 − 3.1)^*∗*^	1.5 (0.7–3.0)
Emergency surgeon	8	9	1.5 (0.5–4.2)	0.7 (0.2–2.3)
Other healthcare professionals	9	6	2.6(0.9 − 7.7)^*∗*^	1.4 (0.4–4.9)
*Received training*				
No	66	105	1	1
Yes	44	29	2.4(1.4 − 4.2)^*∗*^	2.2(1.1 − 4.4)^*∗∗*^
*Number of NASG available*				
Zero	28	93	1	1
One	39	31	4.2(2.2 − 7.9)^*∗*^	2.7(1.3 − 5.5)^*∗∗*^
Two and above	43	10	14.3(6.4 − 32.0)^*∗*^	14.1(5.7 − 35.0)^*∗∗*^
*General knowledge*				
Poor knowledge	20	80	1	1
Good knowledge	90	54	6.7(3.7 − 12.1)^*∗*^	5.2(2.5 − 10.7)^*∗∗*^
*Total attitude*				
Negative attitude	13	60	1	1
Positive attitude	97	74	6.1(3.1 − 11.8)^*∗*^	2.5(1.1 − 5.7)^*∗∗*^

^*∗*^-*p* value <0.2 and ^*∗∗*^-*p* value <0.05.

## Data Availability

Data is available on reasonable request.

## Abbreviations

COR: Crude odd ratio
MMR: Maternal mortality ratio
NASG: Nonpneumatic antishock garment
OR: Odd ratio
PPH: Postpartum hemorrhage
SPSS: Statistical Package for Social Science.

## Supplementary Materials

Supplementary Materials Supplementary materials like questionnaire and datasets will be provided when the journal requests to submit these materials.
