# Supplementary material for: Knowledge, Utilization, and Associated Factors of Nonpneumatic Antishock Garments for Management of Postpartum Hemorrhage among Maternity Ward Health Care Professionals in South Wollo Zone Health Facilities, Ethiopia, 2021: A Cross-Sectional Study Design
Source: Obstet Gynecol Int. 2023 Jan 14;2023:8247603. doi: 10.1155/2023/8247603 (PMC9867589; doi:10.1155/2023/8247603)
Supplement: Supplementary Materials — like questionnaire and datasets will be provided when the journal requests to submit these materials. [file 8247603.f1.docx]

Information sheet and consent form

Health facility category (√) Hospital Health center

Questionnaire identification number___________ Date of data collection ___________E.C

**Introduction**: How are you? My name is ____________________. I am a data collector for the research entitled of “knowledge, utilization and associated factors of NASG for management of PPH among labor and delivery ward health care professionals in selected South Wollo zone health facilities, North west Ethiopia, 2020”. You are selected to be one of the Participants in the study. Your response will provide valuable information about the issue under this study.

**Title of the research project:** Knowledge, utilization and associated factors of NASG for management of PPH among labor and delivery ward health care professionals in selected South Wollo zone health facilities, North West Ethiopia, 2020

**Name of principal investigator**: Wondimnew Gashaw

**Name of sponsoring organization**: Wollo University

**Purpose of research project:** The aim of this study is to assess Knowledge, utilization and associated factors of NASG for management of PPH among labor and delivery ward health care professionals in selected South Wollo zone health facilities, North West Ethiopia, 2020. The findings of this project could help in designing priority intervention strategies for better utilization of NASG and decrease maternal mortality secondary to PPH.

**Procedure:** There are questions that assess Knowledge, utilization and associated factors of NASG for management of PPH among labor and delivery ward health care professionals. I would like to ask you to give your genuine and honest answers on the questions. If you need clarification please ask me. It will take about 15-25 minutes to finish this questionnaire.

**Benefits and risks:** Participating in this research will have no direct benefits. However, the information you provide will help to assess the factors associated with NASG utilization among labor and delivery ward health care professionals in order to appropriately identify future intervention related to problem to be found. Incentive won’t be provided. Your participation in this study will not involve any risks. If a question makes you feel uncomfortable, you may choose not to answer.

**Confidentiality:** Be sure that the information you will provide will be kept strictly confidential. Your name or any identification won’t be documented. Only the researcher will access to see the answers you give.

**Participation:** Participation in the study is completely voluntary. You have the right to respond or not respond to all or some questions. You can also stop the interview in between if you are not interested. Your participation or refusal to participate will have no any effect on you.

**Person to contact:**

If you have any question you can contact.

Name: Wondimnew Gashaw

Phone No: +251918320872 Email: [wondie72g@gmail.com](mailto:wondie72g@gmail.com) or wondimgasha72@gmail.com

Are you willing to participate in this study?

1. Yes Sign and proceed to the questionnaire

2. No
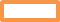
 Thank you!!!

Signature of the respondent -----------------------

Name of data collector--------------------------------------- signature--------------------

Name of supervisor------------------------------------------- signature------------------

Questionnaire

Encircle the responses in the options column if it is a choice question or write their answer on the blank space if it is an open-ended question.

| **Part-I Socio-demographic characteristics of participants** | | | | | | | |
| --- | --- | --- | --- | --- | --- | --- | --- |
| Code | Questions | | | | | Possible responses | Skip to |
|  | How old are you? | | | | | _________years |  |
|  | Sex of respondent? | | | | | 1. Female 2. Male |  |
|  | What is your marital status? | | | | | 1. Single 2. Widowed 3. Divorced 4. Separated 5. Married |  |
|  | What is your religion? | | | | | 1. Muslim 2. Orthodox 3. Protestant   Other(specify)__________ |  |
|  | What is your ethnicity? | | | | | - - - 1. Amhara       2. Oromo       3. Tigray   Other (Specify)_________ |  |
|  | What is your professional qualification? | | | | | 1. Diploma midwife 2. BSc midwife 3. General practitioner 4. Emergency surgeon 5. Gyn/obs specialist   Other (Specify)_______ |  |
|  | How many years of experience do you have as health care professional? | | | | | ____________years |  |
| **Part- II Knowledge on Non-pneumatic Anti-Shock Garment (NASG)** | | | | | | | |
| 1. 2 | Have you ever heard about Non-pneumatic Anti-Shock Garment (NASG)? | | | - - - 1. Yes       2. No | | | 404 |
|  | What is the NASG looks like? | | | 1. Bottom half of suit 2. Gown 3. Trouser | | |  |
|  | What is your first source of information about NASG? | | | 1. Training 2. Health institution as the they used management modality 3. College/ university education 4. Internet   Other specify_________ | | |  |
|  | How many segments does NASG have? | | | 1. Four 2. Six 3. Eight 4. Nine | | |  |
|  | What is/are the function of NASG? (more than one answer is possible) | | | 1. Prevent Shock 2. Stabilize the women in shock 3. Reverse shock 4. Decrease blood loss 5. Compress blood vessels 6. Increase blood flow to vital organs 7. I don’t know | | |  |
|  | What is/are the indications for NASG application? (more than one answer is possible) | | - - - 1. Postpartum hemorrhage (PPH)       2. Shock due to ectopic pregnancy       3. Post cesarean hemorrhage       4. Shock due to trauma with injury/hemorrhage below the diaphragm       5. I don’t know | | | |  |
|  | When NASG applied for women with PPH?  (more than one answer is possible) | 1. Blood loss >750ml 2. Systolic blood pressure <90 mmHg 3. Pulse >110 bpm 4. I don’t know | | | | |  |
|  | What is/are contraindications for use of NASG? (more than one answer is possible) | 1. Viable fetus in-utero 2. Pulmonary edema 3. Bleeding above the diaphragm 4. Congestive heart failure due to mitral stenosis 5. Dyspnea 6. I don’t know | | | | |  |
|  | How to apply NASG? | - - - 1. Start at ankle and proceed up to umbilicus       2. Start at umbilicus then proceed to ankle       3. Start at any segment | | | | |  |
|  | When to remove NASG? (more than one answer is possible) | - - - 1. Estimated blood loss decreased to <50ml/hr       2. Hemoglobin level is >7 or hematocrit is >20%       3. Pulse <100bpm       4. Systolic BP 90mmHg or greater       5. The woman is conscious and aware       6. I don’t know | | | | |  |
|  | What does it mean the rule of 20 cautions for NASG removal? | 1. The time interval between removal of successive segments 2. Blood pressure and pulse which require reapply of segment/s when BP falls by 20 mmHg or pulse increases by 20 bpm respectively. | | | | |  |
| **Part III- Attitude towards Non-pneumatic Anti-Shock Garment (NASG)** | | | | | | | |
| 1. 3 | The use of non-pneumatic anti shock garment is necessary for the management of postpartum hemorrhage in all settings. | | | | - - - 1. Strongly disagree       2. Disagree       3. Neutral       4. Agree       5. Strongly agree | |  |
|  | NASG can be used along with standard treatment protocols of postpartum hemorrhage. | | | | - - - 1. Strongly disagree       2. Disagree       3. Neutral       4. Agree       5. Strongly agree | |  |
|  | NASG can be applied with minimum procedures in short period of time. | | | | - - - 1. Strongly disagree       2. Disagree       3. Neutral       4. Agree       5. Strongly agree | |  |
|  | Removal NASG requires a lot of procedures that takes time. | | | | - - - 1. Strongly disagree       2. Disagree       3. Neutral       4. Agree       5. Strongly agree | |  |
|  | Anti-shock garment is only beneficial to people in the rural areas/primary care settings. | | | | - - - 1. Strongly disagree       2. Disagree       3. Neutral       4. Agree       5. Strongly agree | |  |
|  | Manual removal of placenta is possible with NASG in place. | | | | - - - 1. Strongly disagree       2. Disagree       3. Neutral       4. Agree       5. Strongly agree | |  |
|  | Anti-shock garment is effective in patients with cervical lacerations. | | | | - - - 1. Strongly disagree       2. Disagree       3. Neutral       4. Agree       5. Strongly agree | |  |
|  | The garment should be a must in every health care facility that has maternity service. | | | | - - - 1. Strongly disagree       2. Disagree       3. Neutral       4. Agree       5. Strongly agree | |  |
|  | The garment is only meant to be applied by health care professionals | | | | - - - 1. Strongly disagree       2. Disagree       3. Neutral       4. Agree       5. Strongly agree | |  |
|  | The garment can transmit HIV to patients; hence it is not advisable to be used in a hospital setting. | | | | - - - 1. Strongly disagree       2. Disagree       3. Neutral       4. Agree       5. Strongly agree | |  |
| **Part- IV Utilization of Non-pneumatic Anti-Shock Garment (NASG)** | | | | | | | |
|  | Have you received training on the use NASG? | | | | - - - 1. Yes       2. No | |  |
|  | Does NASG available in your health facility? | | | | - - - 1. Yes       2. No       3. I don’t know | | 404 |
| 403. | If yes to Q 402, how many NASGs available in your health facility? | | | | ________(in number) | |  |
| 404. | Have you ever used NASG in the management of PPH? | | | | - - - 1. Yes       2. No | |  |
| 405. | If no to Q 404, what is/are the reason/s for not utilize NASG for PPH? (more than one answer is possible) | | | | - - - 1. Availability of other method       2. Effective management of third stage of labor       3. Lack of experience       4. Don’t know about NASG       5. The garment is not available | |  |
| 406. | If you know how to use it, will you use it? | | | | - - - 1. Yes       2. No | |  |
| 407. | If it is available, will you use it? | | | | - - - 1. Yes       2. No | |  |

Thank you for your participation!!!
